# The application of stable carbon and nitrogen isotopes to assess the feeding ecology of long-finned pilot whales *(Globicephala melas)* in Scotland

**DOI:** 10.1371/journal.pone.0346340

**Published:** 2026-04-29

**Authors:** Anna Sophie Kebke, Tessa Plint, José Antonio Canseco, Nicholas J. Davison, Mariel T.I. ten Doeschate, Sascha K. Hooker, Clayton R. Magill, Silvia S. Monteiro, Rona McGill, Jason Newton, Clive Trueman, Andrew Brownlow

**Affiliations:** 1 Scottish Marine Animal Stranding Scheme, School of Biodiversity, One Health and Veterinary Medicine, College of Medical, Veterinary and Life Sciences, University of Glasgow, Glasgow, United Kingdom; 2 Centro Oceanográfico de Cádiz (COCAD-IEO), Muelle de Levante, Puerto Pesquero, Cádiz, Spain; 3 Sea Mammal Research Unit, Scottish Oceans Institute, School of Biology, University of St Andrews, St Andrews, United Kingdom; 4 The Lyell Centre for Earth and Marine Sciences, Heriot-Watt University, Edinburgh, United Kingdom; 5 Centre for Environmental and Marine Studies (CESAM) & Department of Biology & ECOMARE/CPRAM, Universidade de Aveiro, Aveiro, Portugal; 6 National Environmental Isotope Facility, Scottish Universities Environmental Research Centre, Glasgow, United Kingdom; 7 Ocean and Earth Science, University of Southampton, Southampton, United Kingdom; Alaska Pacific University, UNITED STATES OF AMERICA

## Abstract

Knowledge of the feeding habits of cetaceans is critical to the development of system-wide conservation strategies in marine ecosystems, yet dietary data are often lacking. To investigate the foraging ecology of long-finned pilot whales (*Globicephala melas*), we analysed stable carbon and nitrogen isotopes in skin tissue from 50 adult and juvenile animals that mass stranded in July 2023 on the Isle of Lewis, Scotland, in the Northeast Atlantic. We interpreted our isotope data with reference to published data from six delphinid species and baseline prey data from the region. We compared isotopic niche breadth among delphinid species and estimated dietary composition for long-finned pilot whales. The average isotopic values were —17.4 ± 0.9 ‰ for *δ*^13^C and +11.0 ± 0.7 ‰ for *δ*^15^N. The core isotopic niche of long-finned pilot whales overlapped with striped dolphin only, with a core niche overlap of 8.6%, suggesting some shared habitat and low trophic level prey, or foraging in habitats with lower baseline *δ*^15^N values. Adult male and female long-finned pilot whales showed complete isotopic niche overlap, although females displayed a wider niche. Estimated dietary contributions suggest a primarily benthopelagic foraging strategy linked to continental shelf edge and slope food webs. Our findings demonstrate the importance of deep-water prey resources to long-finned pilot whales, providing valuable insights into the early spring-summer feeding habits of the species and build baseline ecological data for the Northeast Atlantic. These results highlight the value of stable isotope analysis to advance our understanding of cetacean trophic ecology and better inform marine mammal conservation management.

## 1. Introduction

Recognising and monitoring the feeding habits of cetaceans is key to assessing their role in ecosystem function and stability [1,2] and in developing policies that effectively protect these marine predators. Direct observation of the trophic dynamics of highly mobile cetaceans is however logistically challenging, due to their cryptic nature, inaccessible habitats, and sampling live individuals for indirect diet analysis via tissue biopsy also raises welfare concerns. Although stranding events are sometimes linked to acute or chronic physiological conditions, stranded individuals can serve as valuable representatives of the at-sea living population and provide a cost-effective means of both monitoring and surveillance [3]. Whilst stomach content analysis of a stranded cetacean offers insight into feeding preferences, it has limitations associated with rapid digestion and identifiability of prey remains, such that stomach content remains are often biased toward hard parts and the most recent meal, and hence may misrepresent longer-term diet contributions [4–7]. In instances of live stranding the cetaceans may have vomited due to stress or underlying health conditions [8]. Indirect chemical dietary tracers, most notably fatty acid and stable isotope markers, are a proven tool in cetacean feeding ecology [6,9–11], providing insights at varying timescales [12].

The key principle underlying stable isotope analysis in marine mammal ecology relies on the natural isotopic variation of elements such as carbon and nitrogen being incorporated into the tissue of a consumer via their diet, with the ratios of heavier to lighter isotopes providing important information over various time scales depending on tissue turnover rates [9,10,12,13]. Natural abundance stable isotope ratios of carbon and nitrogen (expressed as *δ*^13^C and *δ*^15^N values, respectively), are the most commonly employed diet tracers in marine mammal ecology. In marine systems, variance in *δ*^13^C values is most strongly associated with variations in fractionation of carbon isotopes during photosynthetic carbon fixation, typically associated with phytoplankton growth rates, temperature and nutrient availability [14–17]. Isotopic spacing between consumers and prey is typically relatively small for carbon (around 1–2 per mille (‰)), consequently *δ*^13^C values in marine consumer tissues can help distinguish feeding and foraging behaviours (e.g., offshore versus inshore) of marine predators by reflecting the baseline carbon sources at the base of the food web. For instance, coastal and benthic primary producers typically exhibit higher *δ*^13^C compared to pelagic sources, owing to the differences in uptake and fractionation of *δ*^13^C [18–20].

The *δ*^15^N values in marine systems vary due to their differences in the isotopic composition of nitrogen sources to primary producers, and due to systematic preferential loss of the light ^14^N isotope in waste metabolites, leading to the well established c. 3–5 ‰ enrichment of *δ*^15^N values in consumer tissues compared to diet [14,21–23].

Both *δ*^13^C and *δ*^15^N values increase with increasing trophic distance from primary production, resulting in systematic increases in consumer isotope compositions with depth, particularly in benthic food webs [24]. The combination and integration of *δ*^13^C and *δ*^15^N analyses in cetacean tissue can be a powerful tool to distinguish between habitat use (coastal, benthic and pelagic, shallow versus deep feeding), as well as assessing an animal’s trophic position and diet composition, providing important insights into ecosystem dynamics and species behaviour [9,10]. As such, stable isotope analysis enables the description of different dimensions of ecological niches in cetacean communities, and identifying overlap and segregations can significantly contribute to our understanding of feeding ecology and food web dynamics [25–27].

In UK waters, more than 20 species of cetaceans occur in coastal and offshore waters, including the long-finned pilot whale (*Globicephala melas*), with 28 species recorded overall [28]. These animals co-exist across feeding guilds and navigate interspecific competition for resources whilst still maintaining a stable population size [29]. As such, understanding where the long-finned pilot whale ecological niche sits within the cetacean community is important, particularly with on-going climate change impacting Northeast Atlantic cetacean populations and their prey [30–32]. Long-finned pilot whales are broadly distributed in coastal and oceanic waters in the west and east North Atlantic, at latitudes of 35–65° N in the western part, and 40–75° N in the eastern part [33–35]. They are highly social and wide-ranging odontocetes with distribution patterns closely linked with the availability and distribution of their primary prey [35–37]. In the Northeast Atlantic, prey is reported to consist mainly of diverse assemblages of deep-water cephalopods [37,38] and fishes (such as greater argentine *(Argentina silus*), Atlantic cod *(Gadus morhua)* and mackerel *(Scombrus scombrus)*) [36,39]. These estimates are based on stomach contents from dead individuals, although this poses interpretative challenges due to the prolonged persistence of squid beaks compared to softer prey remains. Consequently, there is a value in utilising alternative methods – such as stable isotope analysis – that more accurately reflects dietary composition.

Model-based abundance estimates in the Northeast Atlantic have found that long-finned pilot whale distribution in the summer peaks in locations with water depths over 1000 metres, with a distribution strongly associated with the 2000 metre depth contour and areas of moderate slope [40], particularly in areas of high primary productivity [41]. Tagging studies have suggested deep-water bouts of foraging to depths up to 800 metres [42–44]. This is consistent with models from the western North Atlantic, predicting the highest density of long-finned pilot whales along the continental slope [34], consistent with prior reports in the area [33]. As such, common habitats for the species are the continental shelf break, slope waters and areas of high topographic relief, with seasonal movements likely related to prey distributions [33,45]. In winter, species distribution is more southward and in deeper waters [33,34,40,46].

We employed stable carbon and nitrogen isotope analysis to determine the inter-and intraspecific dietary niche of long-finned pilot whales stranded in Scottish waters. Using a 2023 mass stranding event comprised of 55 animals with 54 mortalities, we minimised biases related to the cause of death, as all individuals stranded simultaneously (July) in the same location, and were deemed to be in good health [47]. This provided a unique opportunity to analyse representative individuals from the at-sea population, as long-finned pilot whales in the North Atlantic show high connectivity and low genetic diversity over their entire range [48,49]. In addition, kinship analysis of the samples analysed here found multiple maternally unrelated pod units, and the genotypes present were representative of genotypes found across the eastern North Atlantic over a 20-year period (R. Ball, personal communication, 26 January 2026). We selected skin of adult and juvenile individuals as a tissue with a known and relatively rapid turnover time and hence considered to integrate the assimilated diet over a 2–3 month period for carbon and 3–8 months for nitrogen [50,51].

We used the skin tissue isotope data to ***(a)*** estimate the feeding habitat and estimated diet of long-finned pilot whales inhabiting Northeast Atlantic Scottish waters during the spring-summer months **(*b)*** assess the size and position of the isotopic niche of long-finned pilot whales within the wider delphinid community in Scottish waters and **(*c)*** estimate the contribution of multiple potential prey groups to diet of long-finned pilot whales.

## 2. Materials and methods

### 2.1. Sample collection and preparation

A standardised strandings sampling protocol [52] was undertaken on 54 deceased pilot whales that mass stranded in July 2023 by the Scottish Marine Animal Stranding Scheme (SMASS; NatureScot License Nr. 187976) (Table 1, S1 Table). Age category was assigned based on length values from Bloch *et al.*, 1993. [53], and sex was verified by gross examination of the reproductive organs. To avoid influence from nursing signals seen in calves [9,54], juveniles under 226 cm in body length were deemed to be maternally dependent (*n* = 4) and were excluded from the study. Skin tissue (*n* = 50) sampled cranioventrally to the dorsal fin was stored frozen at −20 °C prior to analysis from which approximately 1 cm^2^ of skin was sub-sampled, desiccated at 60 °C, powdered, and separated into two aliquots. Following a modified Bligh and Dyer (1959) method, one aliquot underwent a lipid extraction process (three rinses of 30 minutes each in 2:1 chloroform:methanol) prior to drying at 60 °C overnight. The paired aliquots underwent no lipid extraction to avoid any deleterious effect of chemical extraction on collagen amino acids and their resultant *δ*^15^N values (Smith et al., 2020). Samples were weighed into pressed tin capsules with a target weight of 0.8 mg. Two outliers with known skin contamination from the stranding and subsequent sampling process (i.e., sand or other organic material) were eliminated from the data analysis (animals M371.21 and M371.34).

**Table 1:** Demographics for long-finned pilot whales (*Globicephala melas)* skin samples collected at the mass-stranding event on the Isle of Lewis, Scotland, in July 2023.

Age	Sex	*n*	Length (cm)
Juvenile	Male	6	227–376
Juvenile	Female	5	226–300
Adult	Male	10	377–602
Adult	Female	29	301–472

#### 2.1.2. Stable isotope analysis.

The relative abundance of carbon (^13^C/^12^C) and nitrogen (^15^N/^14^N) (*δ*^13^C and *δ*^15^N) isotopes were measured using a Thermo Fisher Scientific (TFS; Waltham, MA, USA) IsoLink Elemental Analyser coupled to a TFS Delta V Plus isotope ratio mass spectrometer, using helium as the carrier gas at the National Environmental Isotope Facility (NEIF) Stable Isotope Ecology Laboratory at the Scottish Universities Environmental Research Centre (SUERC). The results of the isotopic analyses are presented in the conventional *δ*-notation [55,56] in per mil (‰) relative to international standards calibrated to VPDB and AIR. Replicate measurements of internal laboratory standards (CNS1.1, *n* = 84) indicated measurement errors of SD 0.16 and 0.17 ‰ for *δ*^13^C and *δ*^15^N, respectively. The carbon to nitrogen (C:N) mass elemental ratio is a commonly used indicator in stable isotope analyses to assess lipid content in biological tissues. Lipids are depleted in ^13^C relative to proteins, and as such, their presence can influence *δ*^13^C values in cetacean skin [57]. In this study, the mean C:N mass ratios for long-finned pilot whale skin were within the expected ranges for cetacean skin (approximately ≤ 4) [9,58–60].

A subset of samples (*n* = 10) were separated into two aliquots to determine tissue lipid content and create a mathematical correction factor for the remaining samples to account for the impact of isotopically light lipid C on analysed skin *δ*^13^C values (McConnaughey and McRoy, 1979; Post et al., 2007). The equation used for this calculation was the following, with the intercept generated being *α* = 7.61 and *β* = 2.33:


$$\delta^{\text{13}}C\;(normalized)\;=\;\delta^{\text{13}}C\;(untreated)\;\;-\;\text{3}.\text{32}\;+\;\text{0}.\text{99}\;x\;C:N$$

#### 2.1.3. Data analysis.

Data analyses were performed using R version 4.2.1 [61]. Data distribution was assessed using a Shapiro-Wilk normality test and Welch’s Two Sample t-test (assuming unequal variance) was used to assess intraspecific differences within the long-finned pilot whale group based on sex and age.

#### 2.1.4. Isotopic niche analysis (SIBER).

We used the SIBER package (Stable Isotope Bayesian Ellipses in R) [62] to calculate intra-specific isotopic niche dimensions within the 2023 mass stranded long-finned pilot whale group. We compared these to the isotopic niches of other delphinid species stranded in the Northeast Atlantic (reported by Plint et al., 2023). To compare isotopic niches, we calculated three metrics: a) Total Area (TA), b) Standard Ellipse Area corrected for small sample sizes (SEA_c_) and c) the Bayesian Standard Ellipse Area (SEA_b_) [63]. TA represents the whole isotopic niche (encompassing 100% of the data) but is less reliable for understanding the core niche. In contrast, SEA_c_ measures the core isotopic niche area (40% of the data) and thus provides an estimate of trophic niche whilst correcting for small sample sizes. In instances where sample sizes are small or uneven, such as that of the age and sex distribution within the pilot whale group, SEA_b_ measures the area of the ellipse but accounts for uncertainty in the data using a Bayesian framework [62]. As a result, SEA_b_ generates posterior distributions of ellipse areas, allowing for estimation of the mode with 95% credible intervals.

#### 2.1.5. Dietary analysis (MixSIAR).

The proportional contributions of different prey sources to the diet of the long-finned pilot whales was estimated using the MixSIAR package [64]. The fundamental principle of MixSIAR is that the isotopic composition of a consumer’s tissue reflects a mixture of its dietary sources (adjusted for isotopic enrichment), with the model assuming that the isotopic values of the consumer fall within the convex mixing space defined by its potential prey. Using Bayesian inference, the probability distribution of dietary proportions that could have produced the observed isotopic values of the consumer is estimated. For all models, chain length was of 100 000, burn-in of 50 000, thinning of 50 and 3 Markov Chain Monte Carlo (MCMC) chains.

Due to global marine isotopic baseline variation [65–67], a regionally specific isotopic prey baseline was compiled for potential prey species collected from UK waters, based on available literature and published data. The isotopic data for each prey species were obtained from the muscle of specimens of fish primarily collected by Trueman *et al.*, (2014) and of squid collected by Monteiro *et al.*, (2015) from waters off western Scotland, and where needed, complemented with data from Jennings and Cogan, 2015 taken from the wider northeast Atlantic shelf seas (a combination of the Celtic Sea, North Sea, Irish Sea and the Channel). The Jennings and Cogan (2015) *δ*^13^C values were mathematically lipid corrected using the equation by Post *et al.*, 2007, whereas the Trueman et al., (2014) and Monteiro et al., (2015) had already been chemically lipid extracted in the original samples. All prey *δ*^13^C source data were Suess corrected to 2023 using the SuessR package [68] to account for the effect of the increase in ^13^C in atmospheric CO_2_ through the burning of fossil fuels [69].

To somewhat account for diet-to-skin enrichment factors caused by biochemical processes, we applied a trophic enrichment factor (TEF) obtained for skin from controlled feeding experiments on bottlenose dolphins (*Tursiops truncatus)* reported by Giménez *et al.*, 2016 due to the close taxonomic relationship between bottlenose dolphins and long-finned pilot whales. As such, in the absence of available TEFs for long-finned pilot whales, we made the assumption that the TEFs would be similar to that of bottlenose dolphins. The SDs for the TEFs reported by Giménez *et al*., 2016 were also included in the model to account for uncertainty. Using these TEFs, we tested two sets of models: a) Model 1, with uninformed priors, b) Model 2, with informed priors (Table 2). As the TEFs were developed using a different species than the one studied here, sensitivity analyses was also conducted for *δ*^13^C and *δ*^15^N under the assumption of different metabolic pathways (e.g., variation in diet quality and composition, growth rate, age, and nutritional status [70–74]) influencing the bulk *δ*^13^C and *δ*^15^N values, that would differentiate the carbon and nitrogen isotopic distribution within the consumer tissue by adjusting the reported TEFs by up to ± 1 σ (1 σ = 0.37 ‰) (S2 Table, S1 Fig). Priors for Model 2 were constructed from dietary information held in the Scottish Marine Animal Stranding Scheme database from 18 long-finned pilot whales stranded on the Scottish coast between June 2011 to July 2015, reporting a total prey number of 25 individual prey items. To account for the intrinsic variability of the data, moderately informed priors were calculated using the formula published by Stock *et al.*, 2018.

**Table 2:** Summary of trophic enrichment factors (TEFs) for the isotopic difference between lipid extracted skin and lipid extracted prey muscle tissue (reported by Giménez *et al.,* (2016)), and the type of uninformed and informed priors used in MixSIAR models for dietary analysis of long-finned pilot whales in the Northeast Atlantic.

	TEFs	TEFs Reference	Priors
**Model 1**	Δ^13^C = 1.01 ± 0.4 Δ^15^N = 1.57 ± 0.5	Giménez *et al.*, (2016)	Uninformed (4 sources)
**Model 2**	Δ ^13^C = 1.01 ± 0.4 Δ^15^N = 1.57 ± 0.5	Giménez *et al.*, (2016)	Informed (4 sources)

The isotopic values of all potential prey items, or representative members of functional prey groups (based on the ecology of the species), were initially plotted on a SIBER plot to visualize the core isotopic niche space of potential prey species in the Northeast Atlantic (S2 Fig). This served as a visual tool to assess isotopic variation among prey groups and informed decisions on which species to include as sources in the subsequent mixing models. For these prey groups, we also visually examined the resulting isotope mixing polygons in the mixing model to ensure that isotopic consumer data fell within the mixing space [75].

**Fig 1:**
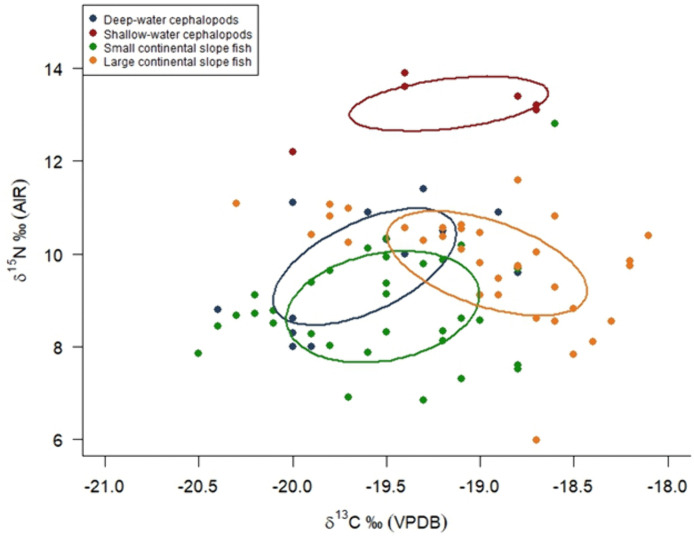
Distribution of potential prey groups representing different feeding habitats, used in the MixSIAR model*δ*^13^C and *δ*^15^N values from Trueman *et al.,* (2014) and Monteiro *et al.,* (2015). Fish and cephalopod muscle tissue *δ*^13^C have been normalised for lipid content following Post *et al.,*(2007) and Suess effect-corrected to 2023. Ellipses contain 40% of the data per species.

Preliminary MixSIAR models using isotopic values from potential prey groups deemed certain prey groups unlikely to represent plausible dietary sources for the population (e.g., Atlantic cod, *Gadus morhua*, and Atlantic mackerel, *Scombrus scombrus*). For the final MixSIAR model, we focused on potential prey species that occupied lower to intermediate trophic levels, falling within the expected isotopic range of the consumer, as well as squid, as these had previously been reported in stomach content analysis of long-finned pilot whales stranded in Scotland between 2011–2015 (G. Pierce, personal communication, 28 May, 2025). Given the inherent limitations of a mixing system with multiple tracers, where increasing number of sources in a two tracer model also increases the model uncertainty [64], we assigned the number of sources in our model through type of prey group based on ecological knowledge on taxonomic group, size and foraging habitat (Table 3; Fig 1).

**Table 3:** Summary of isotopic composition for regionally specific potential prey items used in the MixSIAR model to estimate the diet of long-finned pilot whales stranded on the Isle of Lewis, Scotland, in July 2023.

Functional prey group allocation in MixSIAR model	Functional group	Representative Species	*N*	Mean *δ*^13^C ± SD (‰^2^)	Mean *δ*^15^N ± SD (‰^2^)	Stable isotope data source
**A**	Deep-water cephalopods	*Todarodes sagittatus + Histioteuthis sp.*	13	—19.6 ± 1.2	9.71 ± 1.2	Monteiro et al., (2015)
**B**	Shallow-water cephalopods	*Todaropsis eblanae + Loligo forbesi*	6	—19.1 ± 0.5	13.2 ± 0.6	Monteiro et al., (2015)
**C**	Small continental slope fish (< 30 cm)	*Xenodermichtys copei*	33	—19.52 ± 0.5	8.86 ± 1.2	Trueman et al., (2014)
**D**	Large continental slope fish (> 30 cm)	*Argentina silus*	35	— 18.55 ± 0.5	9.8 ± 1.1	Trueman et al., (2014)

Finally, to avoid potential influences from weaning signals in juvenile individuals in the MixSIAR model, we limited our analyses to adult individuals only. We followed a multiple model approach as suggested by Burnham and Anderson (2004) by fitting and comparing four different models for Models 1 and 2: a) a full model which included length and sex effects, b) a model with sex as a fixed c) a model including only length as continuous covariates, and (d) a null model (no length or sex). Model selection was performed by comparing the models tested based on the leave-one-out cross-validation (LOO) and each model weights [76,77].

## 3. Results

### 3.1. Intraspecific isotopic variation

**Fig 2:**
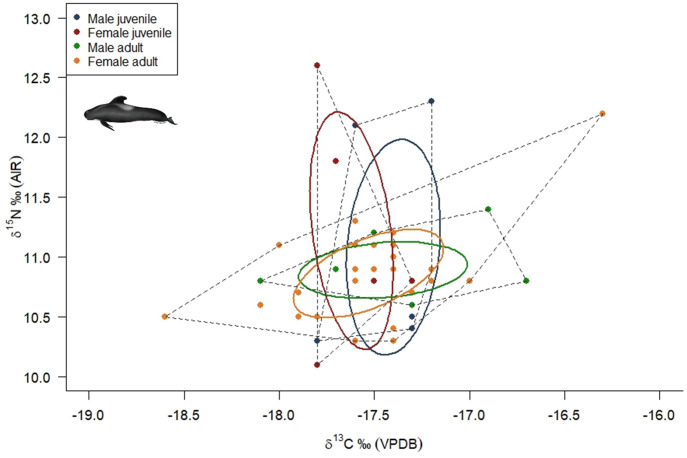
Core (represented by Standard Ellipse Area, containing 40% of the data per group) and total isotopic feeding niches (represented by dashed lines) for Scottish long-finned pilot whale male juveniles (blue circles), female juveniles (red circles), male adults (green circles) and female adults (yellow circles), highlighting differences in isotopic niche size and overlap between sexes and ages.

Here, the mean values for long-finned pilot whale skin were *δ*^13^C = —17.4 ± 0.9 ‰ and *δ*^15^N = +11.0 ± 0.7 ‰ for the whole group, including juveniles and adults (Fig 2). Male animals in the group (body length = 227–602 cm, mean *δ*^13^C —17.3 ± 0.9 ‰ and *δ*^15^N + 11.0 ± 0.7 ‰) were larger on average than females (body length = 226–472 cm, mean *δ*^13^C —17.5 ± 0.4 ‰ and *δ*^15^N + 10.9 ± 0.5 ‰). Within the dataset, no significant difference (Welch’s Two Sample *t*-test) was observed in *δ*^13^C *(t =* — *0.748, p = 0.459)* and *δ*^15^N *(t = −0.505, p = 0.619* between males and female animals*.* The *δ*^13^C and *δ*^15^N values across age groups revealed a general pattern of lower *δ*^13^C values and higher *δ*^15^N values in juveniles compared to adults, particularly in the smaller size range (< 350 cm), with adults showing greater variability in *δ*^13^C compared to *δ*^15^N values, which were more consistent at around +11 ‰. However, no significant differences between adults and juveniles of both sexes were found in *δ*^13^C *(*t *= 0.692, p = 0.495)* and *δ*^15^N *(*t *=* – *0.765, p = 0.459).*

**Fig 3:**
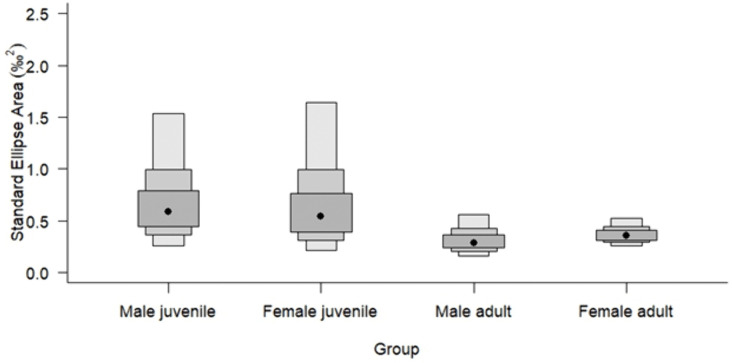
SIBER density plots depicting the niche width (Bayesian standard ellipse areas, SEA_b_) per age and sex group for long-finned pilot whales (*n* *=* *50*) stranded on the Isle of Lewis, Scotland, 2023. Black dots are the mode and shaded boxes represent the 50%, 75% and 95% confidence intervals, reflecting uncertainty in niche width estimates.

Niche analysis revealed that, based on this dataset, adult male and female individuals showed complete niche overlap (Table 4; Figs 2 and 3), with females displaying a broader range of *δ*^13^C (–16.3 to –18.6 ‰) and *δ*^15^N values (+10.3 to +12.2 ‰) (SEA_b_ = 0.36 ‰) compared to males (*δ*^13^C of –16.7. to –18.1 ‰ and *δ*^15^N of +10.6 to +11.4 ‰) (SEA_b_ = 0.29 ‰).

**Table 4:** Mean isotopic values (± SD) and niche areas for age groups of long-finned pilot whales (*n = 50*) including Total Area (TA), Standard Ellipse Area adjusted for small sample size (SEA_c_) and Bayesian Standard Ellipse Area (SEA_b_) mode. All *δ*^13^C values have been lipid-corrected.

Group	*n*	*δ*^13^C ‰ (VPDB) mean ± SD	*δ*^15^N ‰ (AIR) mean ± SD	TA (‰^2^)	SEA (‰^2^)	SEAc (‰^2^)	SEAb (‰^2^) (95% Cl)
**Male juvenile**	6	—17.4 ± 0.2	+ 11.1 ± 0.9	0.82	0.67	0.84	0.61 (0.25–1.54)
**Female juvenile**	5	—17.6 ± 0.2	+ 11.0 ± 0.8	0.74	0.63	0.84	0.56 (0.20–1.65)
**Male adult**	10	— 17.5 ± 0.3	+ 10.9 ± 0.2	0.43	0.31	0.35	0.29 (0.16–0.58)
**Female adult**	29	— 17.2 ± 1.1	+ 11.0 ± 0.7	4.74	0.36	0.38	0.36 (0.26–0.54)

### 3.2. Interspecific isotopic niche overlap with other delphinid species

**Fig 4:**
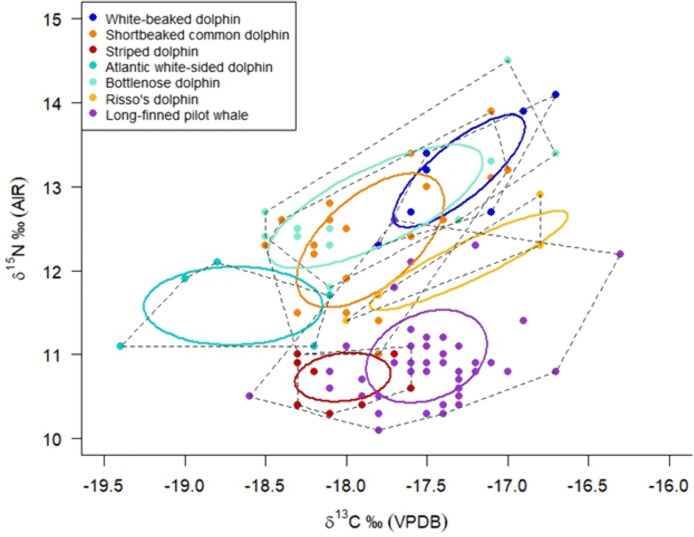
Core (represented by Standard Ellipse Area, containing 40% of the data per group) and total isotopic feeding niches (represented by dashed lines) for six pelagic and deep-diving delphinid species in Scottish waters (data from Plint et al., 2023). The ellipses contain 40% of the data per species, corrected for small sample size [SEA_b_ ‰^2^], including the long-finned pilot whales (purple circles, *n = 50*) from the 2023 Isle of Lewis mass stranding event.

Isotopic niche of the long-finned pilot whales was compared to the pelagic and oceanic delphinid community in Scottish waters [26] (Fig 4). Long-finned pilot whales occupy a distinct isotopic feeding niche within the Scottish pelagic delphinid community and exhibit niche overlap with the striped dolphin *(Stenella coeruleoalba)* with a core niche overlap of 8.6% (Fig 4). Additionally, the range of *δ*^13^C values of the long-finned pilot whales overlapped with that of four other delphinid species: white-beaked dolphins (*Lagenorhynchus albirostris*), Risso’s dolphins (*Grampus griseus*), bottlenose dolphins (*Tursiops truncatus)* and short-beaked common dolphins (*Delphinus delphis*) within the —18.0 to —17.0 ‰ range. For *δ*^15^N values, the range is the same as the striped dolphin, lying within the + 10.0 to 11.5 ‰ range (Fig 4).

### 3.3. Dietary analysis (MixSIAR)

**Fig 5:**
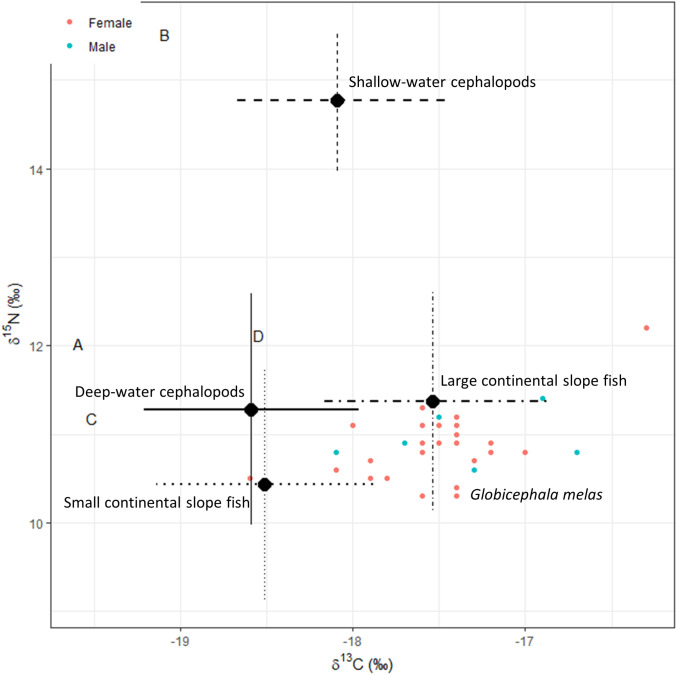
Biplot of *δ*^13^C and *δ*^15^N values of prey (A = deep-water cephalopods, B = shallow-water cephalopods, C= small continental slope fish, D= large continental slope fish) to adult long-finned pilot whales stranded in Scotland, based on skin *δ*^13^C (lipid-corrected) and *δ*^15^N data for females (red) and males (blue). Each point represents the estimated median contribution of a prey group to the diet and error bars indicate 95% credible intervals. Fish data is based on muscle isotope values from Trueman *et al.*, 2014 and cephalopod data is based on mantle isotope values from Monteiro *et al.* 2015. All prey values are corrected for trophic enrichment (TEF) to cetacean skin based on Giménez *et al.* (2016).

**Fig 6:**
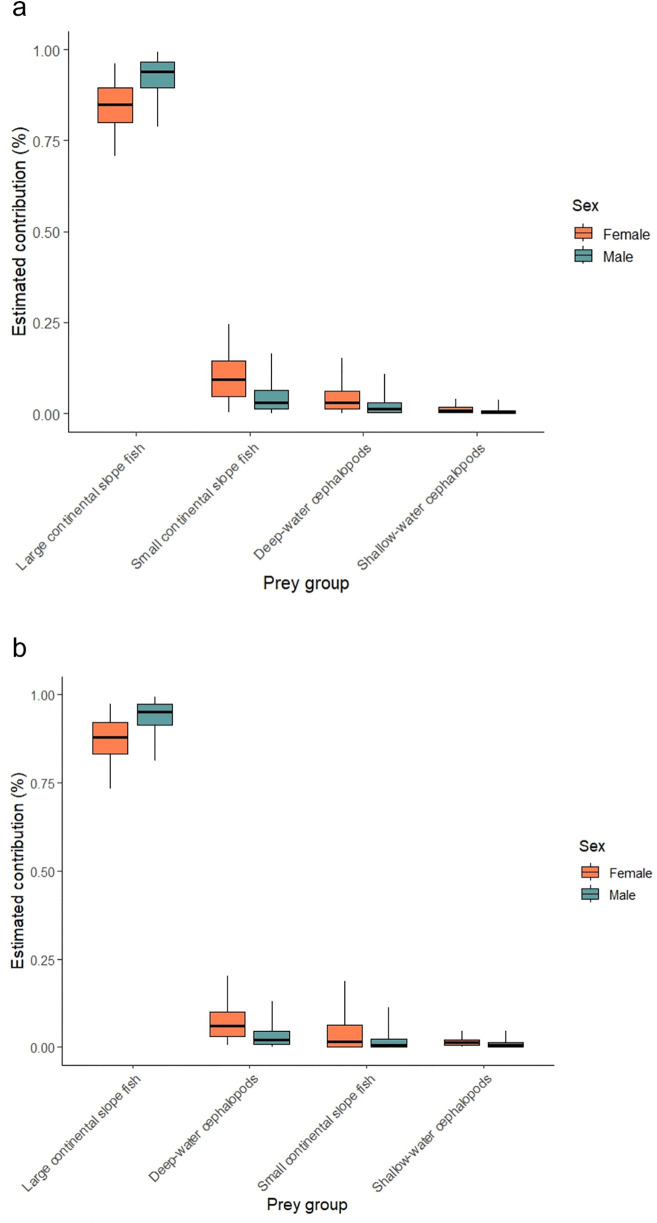
Estimated dietary contributions (median and 95% credibility intervals) of different prey types to female (orange) and male (blue) long-finned pilot whale spring to early summer diet, based on Bayesian mixing models implemented in MixSIAR with an uninformed prior (a) and informed prior (b). Note the x-axis varies between plots.

For both MixSIAR models, visual inspection confirmed that the adult long-finned pilot whale’s isotopic data fell within the expected mixing space for 3 out of the 4 sources, after accounting for the TEFs reported by Giménez *et al.*, 2016. Sensitivity analysis based on TEFs adjusted within the expected range of standard deviation revealed slight differences (S1 Fig, S2 Table). Nevertheless, using the Giménez *et al.*, 2016 reported TEFs (Fig 5), the model that included sex as a fixed effect showed the best predictive performance (Table 5). Model selection based on LOOic weights favoured the sex-influenced model (57% probability with uninformed priors, 50% with informed priors). However, the null model also received support (37% and 47% probability, respectively; Table 5, ΔLOO<2). Under the most informative model (with sex as a fixed effect) with no informative priors (Model 1), the diet of long-finned pilot whales consisted of large fish from the continental slope with a median predicted contribution of 0.84 (84%) (CI_95%_: 0.70–0.96) for females and slightly higher for males at 0.92 (CI_95%_: 0.78–0.98) (Table 6; Figs 5-6). For the most informative model (with sex as a fixed effect) with informative priors (Model 2), the diet also consisted of large fish from the continental slope with a median predicted contribution of 0.87 (87%) (CI_95%_: 0.73–0.96) for females and somewhat higher for males at 0.94 (CI_95%_: 0.81–0.99). Interestingly, small continental slope fish represented the second highest contribution in Model 1 with median values 0.10 (10%) (CI_95%_: 0.004–0.26) for females and 0.04 (4%) (CI_95%_: 0.001–0.16) for males, whereas Model 2 estimated deep-water cephalopods to have the second highest contribution with median values 0.03 (3%) for both females and males (CI_95%_: 0.001–0.18 and 0.001–0.11, respectively). For both models, the contributions of shallow water cephalopods lesser flying squid (*Todaropsis eblanae)* and long-finned squid (*Loligo forbesi)* was almost negligible (Figs 5-6).

**Table 5:** Model comparison for the MixSIAR model with no informed prior (Model 1) and an informed prior (Model 2) using Leave-One-Out Information Criterion (LOOic). Models include combinations of sex and length of long-finned pilot whales as predictors. Δ LOOic represents the difference in LOOic relative to the best-performing model (represented by lowest LOOic), and model weight indicates the relative support for each model among the candidate set.

Model 1 (Uninformed priors)	LOOic	Δ LOOic	Model weight
**Sex only**	16.4	0.0	0.57
**Null (no length or sex)**	17.3	0.9	0.37
**Length only**	21.6	5.2	0.04
**Full (length and sex)**	23.0	6.6	0.02
**Model 2 (Informed priors)**	**LOOic**	**Δ LOOic**	**Model weight**
**Sex only**	16.7	0.0	0.50
**Null (no length or sex)**	16.8	0.1	0.47
**Full (length and sex)**	23.2	6.5	0.02
**Length only**	24.5	7.8	0.01

**Table 6:** Estimated dietary contributions in North-east Atlantic long-finned pilot whales spring to early summer diet by sex based on Bayesian mixing models implemented in MixSIAR with no informed prior (Model 1) and an informed prior (Model 2). Values are median proportional contributions with 95% credibility intervals in parentheses for each prey group: deep-water cephalopods, large continental slope fish, shallow-water cephalopods, and small continental slope fish, prey groups that represent different feeding areas.

Sex	Contribution estimate medians (95% credibility intervals)			
	Model 1 (Uninformed priors)			
	Deep-water cephalopods	Large continental slope fish	Shallow-water cephalopods	Small continental slope fish
**Male**	0.02 (0.0-0.10)	0.92 (0.79-0.99)	0.07 (0.0-0.04)	0.5 (0.001-0.16)
**Female**	0.04 (0.001-0.15)	0.84 (0.71-0.96)	0.01 (0.001-0.04)	0.10 (0.004-0.24)
	**Model 2 (Informed priors)**			
	**Deep-water cephalopods**	**Large continental slope fish**	**Shallow-water cephalopods**	**Small continental slope fish**
**Male**	0.03 (0.001-0.11)	0.94 (0.81-0.99)	0.01 (0-0.04)	0.02 (0-0.08)
**Female**	0.03 (0.001-0.18)	0.87 (0.73-0.96)	0.02 (0.001-0.04)	0.04 (0-0.15)

## 4. Discussion

The 2023 mass stranding of long-finned pilot whales on the Isle of Lewis, Scotland, provided a unique opportunity to investigate the feeding ecology of individuals despite the lack of stomach contents (as revealed by full necropsies). This allowed for comparison without the potential confounds associated with seasonality, differing causes of death, and health status. Necropsy examination indicated good overall health in all individuals prior to stranding, with no viral infections identified, and bacterial infections considered to be within normal limits for the species. Stable isotope analysis of carbon and nitrogen in skin samples of these long-finned pilot whales indicated a primarily benthopelagic feeding strategy. The most informative mixing model indicated a diet dominated by large fish associated with the continental slope, here represented by the greater argentine (*Argentina silus*). Compared to the isotopic niches of other delphinid species stranded in the Northeast Atlantic reported by Plint et al., (2023), only striped dolphins showed core isotopic niche overlap with long-finned pilot whales, indicating shared habitats of continental shelf edge and slope and potential prey overlap. It is possible that there is additional core isotopic niche overlap with other species, such as Risso’s dolphin, but the lack of sufficient isotopic data is a limiting factor in fully understanding this relationship. Whilst acknowledging the biases of sampling only one stranding event, our findings demonstrate that benthopelagic resources are critical to long-finned pilot whales in UK waters, providing key insights into their potential response to changing prey availability and an important consideration for marine mammal management. The long-finned pilot whales’ apparent reliance on shelf-edge and slope habitats, particularly in spring and summer, also highlights priority areas for spatial conservation. Furthermore, this study emphasises the importance of isotopic baseline monitoring using stranded specimens to assess long-term ecological trends in Northeast Atlantic marine mammal populations.

### 4.1. Feeding habitat and isotopic niche of long-finned pilot whales

Mean stable isotope values (*δ*^13^C —17.3 ± 0.9 ‰ and *δ*^15^N + 11.0 ± 0.7 ‰) observed in the skin tissue of the long-finned pilot whales stranded in 2023 indicate feeding within shelf or slope environments, and incorporation of deep water prey (characterised by the moderately enriched ^13^C values compared to pelagic ^13^C values) [9,78,79]. As these long-finned pilot whales stranded in mid-July, their bulk skin *δ*^13^C and *δ*^15^N values incorporate spring and early summer seasonal variation. This is congruent with current knowledge on long-finned pilot whale seasonal distribution as they are known to occupy slope waters during spring to summer months [33,41,46,80], moving to a more offshore and more pelagic lifestyle in winter [33,34,40,46].

Long-finned pilot whale skin samples collected in Scotland between 1992 and 2012 [81], showed higher mean *δ*^13^C values (–18.7 ± 0.7 ‰) relative to this study (17.4 ± 0.9 ‰), while mean *δ*^15^N values were similar (+11.3 ± 0.6 ‰ *versus* +11.0 ± 0.7 ‰, respectively), supporting a consistent trophic position over time. In adjacent Icelandic waters, pooled skin and muscle isotopic analysis from long-finned pilot whale (collected 1988–2021) mean *δ*^13^C values were lower (—18.5 ± 0.3 ‰), with similar mean *δ*^15^N values (11.5 ± 0.3 ‰) to those reported in this study [82]. This might indicate a temporal shift in niche, but equally, these differences may be influenced by variation in lipid extraction methods between the studies. Chemical lipid extraction impacts the original *δ*^15^N values of protein-based tissues, and comparison studies report an increase of 0 to ~2‰ following extraction, although the magnitude of the change varies with the solvent(s) used [57,83–85]. As such, small discrepancies can occur when comparing *δ*^15^N values (e.g., prey values or long-finned pilot whales) between studies that performed a chemical lipid extraction versus a mathematical lipid correction. In Tasmanian waters, Jackson *et al.*, (2025) reported skin *δ*^13^C values –16.6 to – 18.4 ‰ for *δ*^13^C, and +11.3 to + 14.3 ‰ for *δ*^15^N, highlighting similar intraspecific variation to what was observed in this study.

In our dataset, juvenile males exhibited the largest estimated isotopic feeding niche (SEA_b_ 0.61 ‰^2^; SEA_c_ 0.84 ‰^2^), while adult males had the smallest (SEA_b_ 0.29 ‰^2^; SEA_c_ 0.35 ‰^2^). A similar trend was observed in females, with juvenile females showing a broader niche (SEA_b_ 0.56 ‰^2^; SEA_c_ 0.84 ‰^2^) than adult females (SEA_b_ 0.36 ‰^2^; SEA_c_ 0.38 ‰^2^).This trend of broader juvenile niches may reflect limitations in feeding efficiency due to inexperience or physiological constraints related to diving capabilities, but it is more plausible that this variation is caused by residual weaning signal as mixing between maternal milk and independent feeding produces isotopic values that span two distinct dietary sources and/or trophic levels [54,86]. While the one calf with an active nursing signal was excluded from analysis, it is possible that some juvenile skin tissue still retained partial nursing signatures which contributed to the observed variability.

Adult males had smaller SEA_b_ and SEA_c_ values (SEA_b_ 0.29 ‰^2^; SEA_c_ 0.35 ‰^2^) compared to adult females (SEA_b_ 0.36 ‰^2^; SEA_c_ 0.38 ‰^2^), indicating that adult female long-finned pilot whales occupy a broader isotopic niche. This suggests a greater dietary diversity or more varied feeding habitats among female individuals, and is congruent with patterns reported from stable isotope analysis of long-finned pilot whales globally (e.g., [82] (Iceland); [87] (Tasmania)). Further, this may reflect differences in movement patterns between sexes, or links with social foraging behaviour patterns within a pod unit where different pod unit members coordinate their diving behaviour to increase foraging success [44,88].

### 4.2 Isotopic niche overlap with other delphinids

Long-finned pilot whale core isotopic feeding niche overlapped with that of striped dolphin in Scottish waters by 8.6%. The two species showed similar *δ*^15^N values but varied in their *δ*^13^C ranges. This adds another dimension to the findings of Plint *et al.,* (2023), as this study found no interspecific core niche overlap between long-finned pilot whales and five other deep diving and pelagic delphinid species living in Scottish waters. The striped dolphin inhabits the continental shelf and slope out to oceanic waters [89–91] and has been shown to feed on low-level trophic prey, including vertically migrating cephalopods [92] and fish [93]. With the known habitat preferences of long-finned pilot whales [33,34,40], the core niche overlap presented here suggests similar deep-water shelf-edge habitat preferences and some overlap in prey type at a similar trophic level. It is possible that the core isotopic niche overlap would have been larger, had the striped dolphin been sampled during the summer months, as the long-finned pilot whales analysed here. Instead, the striped dolphins analysed by Plint *et al.,* 2023 were sampled across the calendar year, which would have incorporated seasonal dietary signals. Aside from the core isotopic niche overlap, long-finned pilot whale *δ*^13^C values overlapped with that of four other delphinid species reported by Plint *et al.,* (2023), which may either reflect a shared foraging habitat on the continental slope or a reflection of different habitat baselines producing similar isotopic signatures [26,94,[95].

### 4.3 Potential diet contributions

Overall, our findings highlight a significant prey contribution from continental shelf edge and slope food webs to long-finned pilot whale diet. This aligns with previous evidence of the species’ preference for continental shelf breaks and slope waters [33,34,40,45] but not with previously reported mixing model diet estimations for the region, which showed a predominant cephalopod diet [81]. Whilst the contributions of cephalopods were low in our study, deep water cephalopods, European flying squid (*Todarodes sagittatus*) and cock-eyed squid *(Histioteuthis sp.*) contributed more to the diet than shallow water cephalopods, lesser flying squid (*Todaropsis eblanae*) and long-finned squid *(Loligo forbesi)*, which is in accordance with the findings of Monteiro *et al.,* (2015). Our diet estimation suggests a diet mainly composed of large fish inhabiting the continental slope, with greater argentine representing this prey group.

Although greater argentine has previously been identified in the diet of long-finned pilot whales [96], our findings more likely reflect a seasonal foraging preference – spring to early summer – within benthopelagic habitats along the continental slope. Both greater argentine and bluntsnout smooth-head *(Xenodermichtys copei*) are slope-associated fish species, typically found at depths up to 800 metres [97–100]. The substantial dietary contribution of these species (or the habitat and trophic level that they represent) suggests that long-finned pilot whales in this study have been feeding closely along the continental slope or shelf edge during spring to early summer in Northeast Atlantic waters.

The mixing model including sex as a fixed effect was the most informative, further reinforcing potential differences in foraging strategies and prey preferences between males and females. This result also supports the intraspecific isotopic niche results presented in this study, wherein the female animals occupied a broader isotopic niche. The mixing polygon also highlights either a potentially unexplored prey source, distinct from the cephalopod and fish groups included in the mixing models. This potential prey source would likely have values around or lower than +10.5 ‰ for *δ*^15^N and between — 18.0 and — 17.0 ‰ for *δ*^13^C. It is more plausible that the observed isotopic position of the consumers (i.e., the long-finned pilot whales) on the MixSIAR plot (Fig 6) results from incorrect diet-tissue enrichment factors rather than a missing dietary input source. Additionally, the prey values used were not recent, and prey isotopic signatures might have changed over time, not accounting for interannual variability [101]. Our study relied on trophic enrichment factors (TEFs) derived from another delphinid species (bottlenose dolphins) due to the lack of species-specific TEFs for long-finned pilot whales. Consequently, the known physiological differences between these species, including body size, diving capabilities, and metabolic processes, may have led to discrepancies when applying non-species specific TEFs [70,102]. This effect was evident in our study when applying sensitivity analysis (S1 Fig, S2 Table), highlighting the need to better understand metabolic pathways and distributions to more confident interpretations and to differentiate between sources, and that the TEF may be responsible for the discrepancies observed here.

If the discrepancies cannot be attributed to TEFs, we did explore benthic nutrient sources as a possibility for this “missing prey group” within the mixing model. Interestingly, some benthic invertebrates, such as sponges (e.g., of the phylum Porifera) and bristle worms (e.g., of the phylum Polychaeta) in the area have similar isotopic compositions, suggesting a benthic nutrient source may fit the model [103,104]. This theory was initially explored as a possibility but was excluded from the final analysis presented here due to the high uncertainty associated with the diet contribution estimates, likely resulting from the number of sources incorporated in the model. It is nonetheless an intriguing hypothesis that should be investigated further, particularly as there are communities associated with Northeast Atlantic cold-water coral reefs housing a variety of benthic invertebrates [103,104]. In Scottish waters, a notable sponge belt is that of the Faroe-Shetland Channel, with a pronounced continental slope ideal for sponge aggregations to settle at around 350–650 metres deep and with a sedimentary community dominated by polychaete worms [105], north of where the long-finned pilot whales in our study stranded. Whilst an unlikely prey source, the high density of these invertebrates in this area could have been an attractive and easy prey source. However, the complexities of the multifaceted biogeochemical processes driving isotopic incorporation in benthic taxa need to be considered on a local level [106,107] and is outside of the scope of this study. Even so, this intricacy underscores the importance of establishing and utilising regionally and temporally specific baselines when interpreting isotopic data, and the value of collecting and building local isotopic prey libraries.

It is also important to acknowledge the inherent limitations of mixing models when interpreting outputs. These are well described in Phillips *et al.*, 2014, but to summarise, one major challenge encountered here is, once again, the uncertainty around trophic enrichment factors (TEFs), which (unless species-specific) may cause misleading results and may not always be true in systems with nonlinear metabolic pathways, as TEFs vary between species owing differences in metabolism, growth rate, protein turnover and excretion pathways [70]. However, obtaining a species-specific TEF for a large cetacean comes with its own challenges and has ethical considerations, and the R package SIDER, aimed to predict TEFs of consumers based on their ecology and phylogenetic relatedness, currently do not include cetacean data [108]. The model also assumes dietary stability over the sampling window which, for mobile marine predators, may not be valid, and requires enough time for the isotopic signal to incorporate from the prey tissue to the predator. As such, in complex ecological settings such as oceanic ecosystems, interpreting posterior estimates of the model comes with caveats. The results need to be interpreted with caution and analysed together with other pieces of evidence (e.g., faecal eDNA sampling and direct feeding observations), underscoring the importance of multidisciplinary collaboration. Ideally, we would have utilised prey isotopic values taken from the same year and region as that of our consumer samples, which would have minimised any interannual variability, as well as any biases related to sample preservation and pre-treatment of prey samples [101]. Available stomach content data for Northeast Atlantic long-finned pilot whales was scarce and as such, the priors could have been biased towards prey with slower digestion rates (e.g., cephalopods), highlighting the need for more published stomach content data.

### 4.4. Conclusions

This study provides novel insight into the feeding ecology of long-finned pilot whales in the Northeast Atlantic, and specifically Scotland, highlighting their reliance on deep water prey associated with continental shelf edge and slope habitats in the spring to early summer season. Stable isotope analysis of *δ*^13^C and *δ*^15^N in skin tissue revealed a distinct core isotopic niche overlapping only marginally with striped dolphins. Adult male and female long-finned pilot whales showed complete isotopic niche overlap, but female animals displayed a wider isotopic foraging niche, highlighting a broader use of resources. Here, we have generated and updated foundational baseline ecological information for long-finned pilot whales in Scottish waters, which contributes to the evidence base for marine mammal management. Understanding the trophic ecology and dietary preferences of cetaceans is essential for supporting conservation and management strategies, particularly in locations where the impact of anthropogenic activities may pose threats to cetacean populations. When combined with other methods, such as informed stomach content analysis, metagenomics on gastrointestinal or faecal eDNA material, stable isotope analysis offers a powerful approach for reconstructing diet and identifying longer-term foraging patterns that might not be evident from short-term observations alone. Additionally, this study demonstrates the benefits of citizen science data when studying cetacean feeding ecology, as sample collection was supported not only by scientists but also by volunteers and members of the public. We recommend stranding networks request that volunteers collect a small skin fragment specifically for stable isotope analysis, providing a practical and direct pipeline for better understanding of the feeding ecology and diet of a cryptic cetacean species.

To tease apart trophic and baseline contributions to bulk tissue isotopic variation, we recommend future studies apply compound-specific stable isotope analysis of amino acids for finer resolution information on trophic position and nutrient source [109–111]. Whilst we consider our results representative of the at-sea population in Scottish waters due to evidence from genetic analysis pointing toward the limited population structure of the species in the Northeast Atlantic [48,49], we recommend a similar bulk isotope approach to be undertaken with skin samples from the eight other documented long-finned pilot whale mass stranding events in Scotland to date (2011–2024, totalling 198 fatalities) to build a comprehensive spatial and temporal dataset of feeding ecology and dietary habits across two decades. Analysis of *δ*^13^C and *δ*^15^N values from the skin of four long-finned pilot whale mass stranding events in Tasmania showed that stranding location accounted for most of the isotopic variability observed, likely reflecting unique spatial ranges differences in foraging habitats [87]. Assessment of long-finned pilot whales’ trophic ecology collected from 16 strandings in Iceland was used to build a baseline for future studies to determine niche overlap between Icelandic cetaceans [82]. Applying a similar methodology to stranded long-finned pilot whales on the UK coastline could help generate and update baseline ecological data for the species and, by extension, contribute to the evidence base for marine mammal management, including fisheries and conservation strategies under the UK Marine Strategy, OSPAR, and the Habitat Regulations. Together, these findings establish a critical framework for reconstructing the feeding ecology of long-finned pilot whales in UK waters – an essential step toward informed conservation, effective fisheries management, and the long-term resilience of cetacean populations in the Northeast Atlantic.

## Supporting information

S1 Table Age, sex, length, *δ*^13^C and *δ*^15^N values of mass stranded long-finned pilot whales sampled by the Scottish Marine Animal Stranding Scheme (SMASS) on the Isle of Lewis, Scotland, used in this study (n = 50). (DOCX)

S2 Table Trophic enrichment factor (TEF) values for lipid extracted skin and lipid extracted prey muscle tissue by Giménez *et al.,* (2016), here adjusted for sensitivity analysis. (DOCX)

S1 Fig Sensitivity analysis output when trophic enrichment factor (TEF) by Giménez *et al.*, (2016) for *δ*^13^C and *δ*^15^N are adjusted by (a) – 1 σ uncertainty and b) + 1 σ uncertainty. A = deep-water cephalopods, B = shallow-water cephalopods, C = small continental slope fish, D = large continental slope fish), orange dots represent female adult individuals, green dots represent male adult individuals.
(DOCX)

S2 Fig Distribution of potential long-finned pilot whale prey species δ^13^C and δ^15^N. Fish and cephalopod muscle tissue δ^13^C have been normalised for lipid content following Post et al (2007) and Suess-effect corrected to 2023.
(DOCX)
