# Supplementary material for: The application of stable carbon and nitrogen isotopes to assess the feeding ecology of long-finned pilot whales (Globicephala melas) in Scotland
Source: PLoS One. 2026 Apr 29;21(4):e0346340. doi: 10.1371/journal.pone.0346340 (PMC13127942; doi:10.1371/journal.pone.0346340)
Supplement: S1 Table — (DOCX) [file pone.0346340.s001.docx]

**SUPPLEMENTARY MATERIALS: The application of stable carbon and nitrogen isotopes to assess the feeding ecology of long-finned pilot whales *(Globicephala melas)* in Scotland**

**Table S1.** **Age, sex, length, *δ*^13^C and *δ*^15^N values of mass stranded long-finned pilot whales sampled by the Scottish Marine Animal Stranding Scheme (SMASS) on the Isle of Lewis, Scotland, used in this study (n = 50).**

| SMASS Reference number | Skin *δ*^13^C ‰ (VPDB) | Skin *δ*^15^N ‰ (AIR) | Length (cm) | Sex | Age | Pregnancy/lactation status |
| --- | --- | --- | --- | --- | --- | --- |
| M371.1/23 | –17.3 | 10.8 | 300 | Female | Juvenile | No |
| M371.2/23 | –17.4 | 11.0 | 415 | Female | Adult | No |
| M371.3/23 | –17.0 | 10.8 | 406 | Female | Adult | No |
| M371.4/23 | –17.2 | 10.9 | 412 | Male | Adult | N/A |
| M371.5/23 | –17.2 | 10.9 | 390 | Female | Adult | No |
| M371.6/23 | –17.6 | 10.9 | 459 | Male | Adult | N/A |
| M371.7/23 | –17.6 | 10.3 | 391 | Female | Adult | No |
| M371.8/23 | –17.5 | 11.2 | 450 | Male | Adult | N/A |
| M371.10/23 | –16.9 | 11.4 | 602 | Male | Adult | N/A |
| M371.11/23 | –17.3 | 10.5 | 283 | Male | Juvenile | N/A |
| M371.12/23 | –17.5 | 10.9 | 365 | Female | Adult | No |
| M371.13/23 | –17.2 | 10.8 | 430 | Female | Adult | Recent parturition & lactating |
| M371.14/23 | –17.4 | 11.2 | 396 | Female | Adult | No |
| M371.15/23 | –18.1 | 10.6 | 472 | Female | Adult | Recent parturition & lactating |
| M371.16/23 | –17.4 | 11.0 | 385 | Female | Adult | No |
| M371.17/23 | –17.4 | 11.1 | 426 | Female | Adult | No |
| M371.18/23 | –17.8 | 12.6 | 233 | Female | Juvenile | No |
| M371.19/23 | –17.3 | 10.4 | 290 | Male | Juvenile | N/A |
| M371.20/23 | –17.6 | 10.9 | 435 | Male | Adult | N/A |
| M371.22/23 | –16.7 | 10.8 | 376 | Male | Adult | N/A |
| M371.23/23 | –17.6 | 10.9 | 465 | Female | Adult | - |
| M371.24/23 | –17.5 | 11.1 | 419 | Female | Adult | No |
| M371.25/23 | –17.4 | 10.4 | 447 | Female | Adult | Recent parturition & lactating |
| M371.27/23 | –18.1 | 10.8 | 444 | Male | Adult | N/A |
| M371.28/23 | –17.7 | 10.9 | 510 | Male | Adult | N/A |
| M371.29/23 | –17.6 | 10.9 | 400 | Female | Adult | No |
| M371.30/23 | –17.3 | 10.7 | 390 | Female | Adult | No |
| M371.31/23 | –17.2 | 10.9 | 380 | Male | Juvenile | N/A |
| M371.32/23 | –17.6 | 11.3 | 404 | Female | Adult | Recent parturition & lactating |
| M371.33/23 | –17.5 | 10.8 | 258 | Female | Juvenile | No |
| M371.35/23 | –17.9 | 10.5 | 360 | Female | Adult | No |
| M371.36/23 | –17.6 | 11.1 | 398 | Female | Adult | Recent parturition & lactating |
| M371.37/23 | –17.6 | 10.8 | 410 | Female | Adult | No |
| M371.38/23 | –17.6 | 10.8 | 400 | Female | Adult | Recent parturition & lactating |
| M371.39/23 | –17.5 | 10.9 | 426 | Female | Adult | Pregnant |
| M371.40/23 | –17.4 | 10.9 | 450 | Female | Adult | Pregnant |
| M371.41/23 | –17.6 | 10.8 | 277 | Female | Juvenile | No |
| M371.42/23 | –17.8 | 10.5 | 374 | Female | Adult | Pregnant |
| M371.43/23 | –16.3 | 12.2 | 399 | Female | Adult | Pregnant |
| M371.44/23 | –18.1 | 10.8 | 529 | Male | Adult | N/A |
| M371.45/23 | –17.8 | 10.3 | 302 | Male | Juvenile | N/A |
| M371.46/23 | –17.9 | 10.7 | 423 | Female | Adult | Recent parturition & lactating |
| M371.47/23 | –17.3 | 10.6 | 406 | Male | Adult | N/A |
| M371.48/23 | –17.8 | 10.1 | 293 | Female | Juvenile | No |
| M371.49/23 | –18.6 | 10.5 | 384 | Female | Adult | Lactating |
| M371.50/23 | –17.4 | 10.3 | 425 | Female | Adult | Recent parturition & lactating |
| M371.51/23 | –17.6 | 12.1 | 227 | Male | Juvenile | N/A |
| M371.52/23 | –18.0 | 11.1 | 415 | Female | Adult | Recent parturition & lactating |
| M371.53/23 | –17.7 | 11.8 | 226 | Female | Juvenile | No |
| M371.54/23 | –17.2 | 12.3 | 240 | Male | Juvenile | N/A |
