# Supplementary material for: The application of stable carbon and nitrogen isotopes to assess the feeding ecology of long-finned pilot whales (Globicephala melas) in Scotland
Source: PLoS One. 2026 Apr 29;21(4):e0346340. doi: 10.1371/journal.pone.0346340 (PMC13127942; doi:10.1371/journal.pone.0346340)
Supplement: S2 Table — (DOCX) [file pone.0346340.s002.docx]

**SUPPLEMENTARY MATERIALS: The application of stable carbon and nitrogen isotopes to assess the feeding ecology of long-finned pilot whales *(Globicephala melas)* in Scotland**

**Table S2. Trophic enrichment factor (TEF) values for lipid extracted skin and lipid extracted prey muscle tissue by Giménez et al., (2016), here adjusted for sensitivity analysis.**

|  | TEFs (adjusted for sensitivity analysis) | TEFs Reference | Sensitivity analysis |
| --- | --- | --- | --- |
| Model a | *δ*^13^C = 0.60 *δ*^15^N = 1.05 | Giménez *et al.*, (2016) | - 1 σ uncertainty |
| Model b | *δ*^13^C = 1.38 *δ*^15^N = 2.09 | Giménez *et al.*, (2016) | + 1 σ uncertainty |
