# Supplementary material for: The application of stable carbon and nitrogen isotopes to assess the feeding ecology of long-finned pilot whales (Globicephala melas) in Scotland
Source: PLoS One. 2026 Apr 29;21(4):e0346340. doi: 10.1371/journal.pone.0346340 (PMC13127942; doi:10.1371/journal.pone.0346340)
Supplement: S1 Fig — A = deep-water cephalopods, B = shallow-water cephalopods, C = small continental slope fish, D = large continental slope fish), orange dots represent female adult individuals, green dots represent male adult individuals. (DOCX) [file pone.0346340.s003.docx]

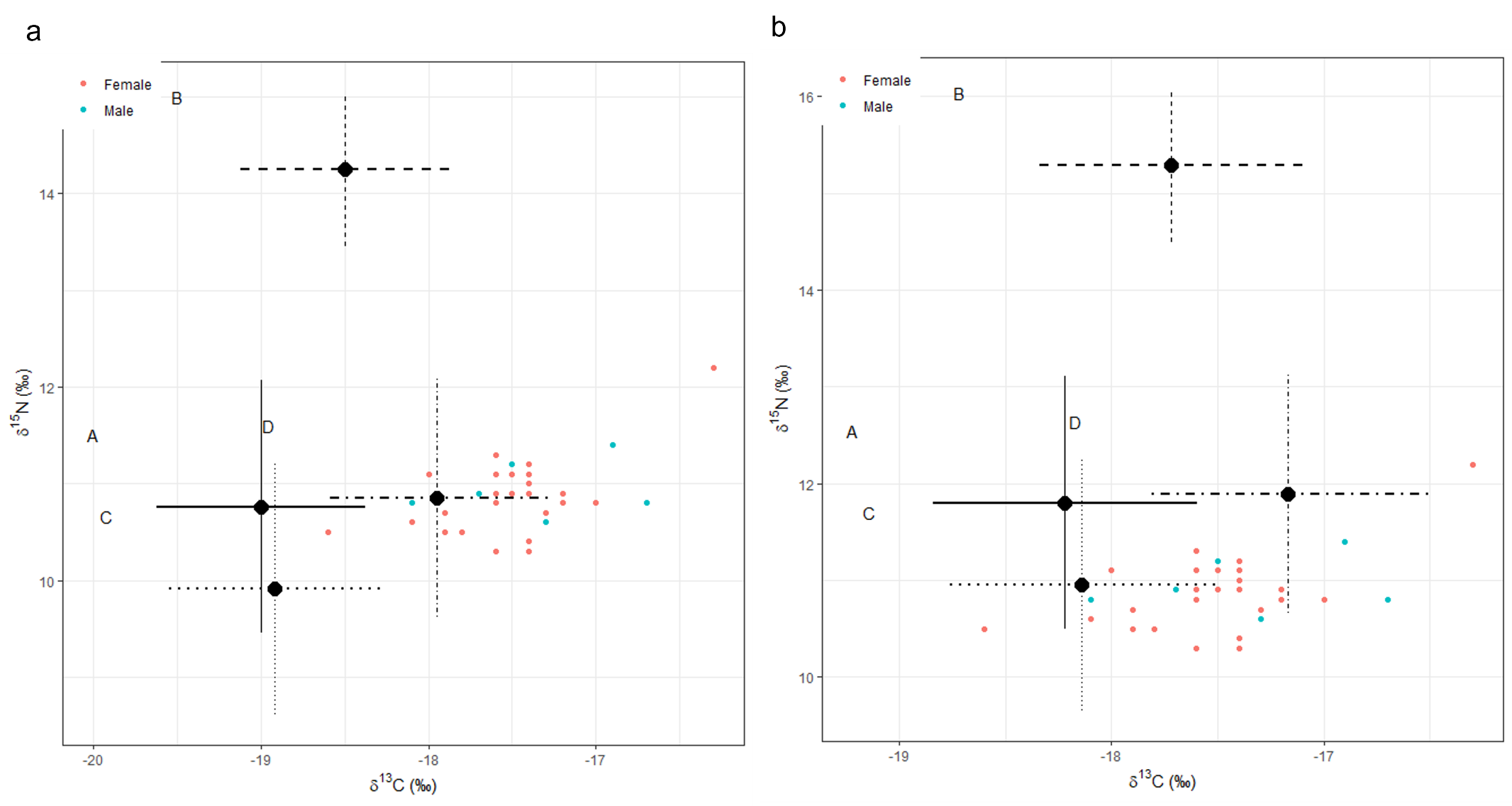


**Figure S1. Sensitivity analysis output when trophic enrichment factor (TEF) by Giménez *et al.*, (2016) for *δ*^13^C and *δ*^15^N are adjusted by (a) - 1 σ uncertainty and b) + 1 σ uncertainty.** **A = deep-water cephalopods, B = shallow-water cephalopods, C = small continental slope fish, D = large continental slope fish**), orange dots represent female adult individuals, green dots represent male adult individuals.
