# Supplementary material for: The application of stable carbon and nitrogen isotopes to assess the feeding ecology of long-finned pilot whales (Globicephala melas) in Scotland
Source: PLoS One. 2026 Apr 29;21(4):e0346340. doi: 10.1371/journal.pone.0346340 (PMC13127942; doi:10.1371/journal.pone.0346340)
Supplement: S2 Fig — Fish and cephalopod muscle tissue δ13C have been normalised for lipid content following Post et al (2007) and Suess-effect corrected to 2023. (DOCX) [file pone.0346340.s004.docx]

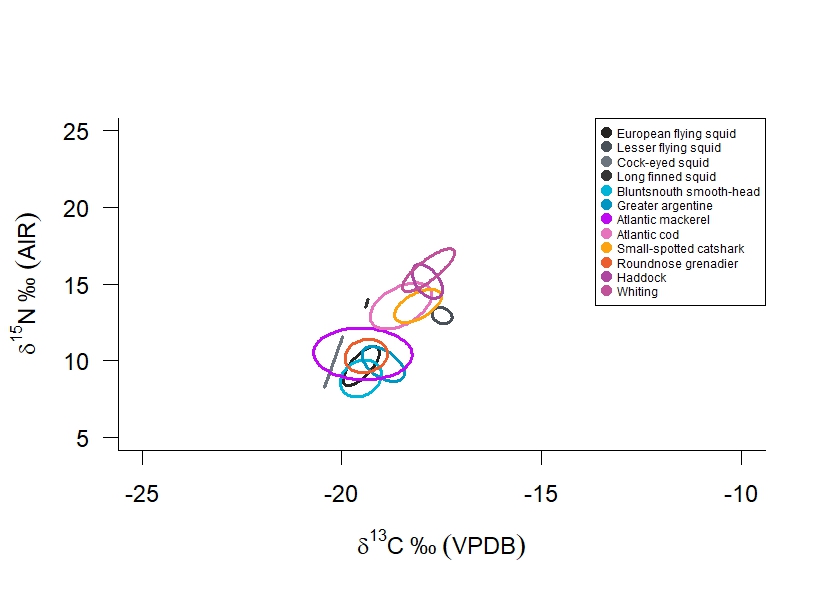


**Figure S2*.* Distribution of potential long-finned pilot whale prey species δ^13^C and δ^15^N.** Fish and cephalopod muscle tissue δ^13^C have been normalised for lipid content following Post et al (2007) and Suess-effect corrected to 2023.
